# Effective nebulization of interferon-γ using a novel vibrating mesh

**DOI:** 10.1186/s12931-019-1030-1

**Published:** 2019-04-03

**Authors:** Louise Sweeney, Alice P. McCloskey, Gerard Higgins, Joanne M. Ramsey, Sally-Ann Cryan, Ronan MacLoughlin

**Affiliations:** 1 Aerogen, IDA Business Park, Dangan, Galway, Ireland; 2 School of Pharmacy, RCSI, Dublin, Ireland; 3 Tissue Engineering Research Group (TERG), RCSI, Dublin, Ireland; 4 Trinity Centre for Bioengineering (TCBE), TCD, Dublin, Ireland; 5 School of Pharmacy and Pharmaceutical Sciences, Trinity College, Dublin, Ireland; 6 Centre for Research in Medical Devices (CÚRAM) NUIG & RCSI, Dublin, Ireland

**Keywords:** Idiopathic pulmonary fibrosis, Tuberculosis, Interferon gamma, Inhaled therapy, Vibrating mesh, Nebulizer, Aerosol

## Abstract

**Background:**

Interferon gamma (IFN-γ) is a clinically relevant immunomodulatory cytokine that has demonstrated significant potential in the treatment and management of respiratory diseases such as tuberculosis and pulmonary fibrosis. As with all large biomolecules, clinical translation is dependent on effective delivery to the disease site and delivery of IFN-γ as an aerosol offers a logical means of drug targeting. Effective localization is often hampered by instability and a lack of safe and efficient delivery systems. The present study sought to determine how effectively IFN-γ can be nebulized using two types of vibrating mesh nebulizer, each with differing mesh architectures, and to investigate the comparative efficiency of delivery of therapeutically active IFN-γ to the lungs.

**Methods:**

Nebulization of IFN-γ was carried out using two different Aerogen vibrating mesh technologies with differing mesh architectures. These technologies represent both a standard commercially available mesh type (Aerogen Solo®) and a new iteration mesh (Photo-defined aperture plate (PDAP®). Extensive aerosol studies (aerosol output and droplet analysis, non-invasive and invasive aerosol therapy) were conducted in line with regulatory requirements and characterization of the stability and bioactivity of the IFN-γ post-nebulization was confirmed using SDS-PAGE and stimulation of Human C-X-C motif chemokine 10 (CXCL 10) also known as IFN-γ-induced protein 10KDa (IP 10) expression from THP-1 derived macrophages (THP-1 cells).

**Results:**

Aerosol characterization studies indicated that a significant and reproducible dose of aerosolized IFN-γ can be delivered using both vibrating mesh technologies. Nebulization using both devices resulted in an emitted dose of at least 93% (100% dose minus residual volume) for IFN-γ. Characterization of aerosolized IFN-γ indicated that the PDAP was capable of generating droplets with a significantly lower mass median aerodynamic diameter (MMAD) with values of 2.79 ± 0.29 μm and 4.39 ± 0.25 μm for the PDAP and Solo respectively. The volume median diameters (VMD) of aerosolized IFN-γ corroborated this with VMDs of 2.33 ± 0.02 μm for the PDAP and 4.30 ± 0.02 μm for the Solo. SDS-PAGE gels indicated that IFN-γ remains stable after nebulization by both devices and this was confirmed by bioactivity studies using a THP-1 cell model in which an alveolar macrophage response to IFN-γ was determined. IFN-γ nebulized by the PDAP and Solo devices had no significant effect on the key inflammatory biomarker cytokine IP-10 release from this model in comparison to non-nebulized controls. Here we demonstrate that it is possible to combine IFN-γ with vibrating mesh nebulizer devices and facilitate effective aerosolisation with minimal impact on IFN-γ structure or bioactivity.

**Conclusions:**

It is possible to nebulize IFN-γ effectively with vibrating mesh nebulizer devices without compromising its stability. The PDAP allows for generation of IFN-γ aerosols with improved aerodynamic properties thereby increasing its potential efficiency for lower respiratory tract deposition over current technology, whilst maintaining the integrity and bioactivity of IFN-γ. This delivery modality therefore offers a rational means of facilitating the clinical translation of inhaled IFN-γ.

## Background

Cytokines, including the interferons (IFNs), play an essential role in immune and inflammatory responses [1]. The IFNs are subdivided into two classes (Type I and Type II) based on their sequence and specificity for particular receptors. Type II includes a single and distinct molecule, IFN-γ which is produced in activated T-cells and natural killer cells in response to interleukin (IL)-12 and IL-18 being produced by antigen presenting cells [2]. These important molecules are involved in the underlying pathophysiology of a number of infectious and inflammatory associated disease states including idiopathic pulmonary fibrosis (IPF), tuberculosis (TB), osteoporosis, chronic granulomatous disease (CGD) and systemic lupus erythematous (SLE) among others [3].

IFN-γ is used clinically in the management of CGD (resulting from leukocyte dysfunction) and severe malignant osteoporosis (a congenital disorder associated with increased bone density due to impaired resorption of osteoclasts). Both conditions result in patients regularly suffering from life threatening infections. Prophylactic treatment with IFN-γ reduces the frequency and relative risk of severe infections and may delay disease progression [4]. Currently, two recombinant IFN-γ products are available; Actimmune® (Horizon Pharma USA, Inc) and Immukin® (Boehringer Ingelheim, Germany). These identical liquid preparations are only licensed at present for the aforementioned conditions and only for parenteral administration [1]. Current research is focusing on alternative applications for IFN-γ, and is largely underpinned by an assumption that therapeutic effects are due to its immunomodulatory properties [5, 6].

Within respiratory medicine there is a growing interest in a potential role for IFN-γ in the treatment of IPF, TB and other inflammatory respiratory conditions [7]. A multinational study of 330 IPF patients showing the subcutaneous administration of IFN-γ versus placebo, concluded that IFN-γ did not affect progression-free survival, pulmonary function, or the quality of life [8]. However, this study also concluded that due to the size of the patient population and trial duration, a clinically significant survival benefit could not be ruled out. Work such as this has led researchers to explore direct, targeted delivery of the biomolecule via inhalation, as an alternative route of administration to injection. There are various aerosol delivery modalities. Aerosols may be delivered via an endotracheal tube. In this modality, the patient will be under direct clinical supervision and therefore in a hospital or clinical setting. Inhalation of aerosol formulations may also via a non-invasive route of administration, e.g. facemask, with the potential for targeted delivery within the lung, and consequent improved clinical response with minimal adverse effects [9]. A study on healthy volunteers comparing parenterally delivered to aerosolized IFN-γ, showed that subcutaneous administration resulted in detectable levels of IFN-γ in serum, but not in epithelial lining fluid (ELF) [10, 11]. There was also evidence of IP-10 expression (a gene induced specifically by IFN-γ) in blood monocytes but not alveolar macrophages in the lungs, in addition to multiple systemic adverse effects. Inhaled IFN-γ on the other hand was not detectable in serum but was detectable in respiratory ELF, in a dose dependent fashion. Alveolar macrophages, but not blood monocytes, expressed IP-10 mRNA transcripts, and delivery via inhalation precipitated negligible local or systemic adverse effects.

Aerosolized IFN-γ has been used in numerous clinical trials in combination with other standard treatments. For example, Moss and colleagues [12] administered Actimmune via a jet nebulizer to CF patients and determined 12 week outcomes. Whilst it was well-tolerated, there was no significant benefit in terms of patient outcomes with inflammatory sputum markers, bacterial load and lung function all remaining the same. Others have also investigated the aerosolisation of IFN-γ for application in both TB and IPF [13]. Increasing resistance to TB therapies is a global medical challenge resulting in complicated, lengthier and more toxic treatment regimens and an ever-limited range of treatment options [14]. A trial of inhaled IFN-γ in multi-drug resistant TB showed that, similar to Moss and colleagues, it was well tolerated [15]. There was also evidence in this study of improvement in symptoms with topography chest scans showing a reduction in cavity lesion size, reduced bacterial loads for acid-fast-bacillus in sputum and *Mycobacterium tuberculosis.*

Nebulizers facilitate drug delivery to the lungs in the form of an aerosolized drug solution or suspension with properties including droplet size distribution of the emitted aerosols and output rate considered key to aerosol generator performance and ultimate drug deposition profiles within the lungs [16, 17]. Nebulizers can be very beneficial in treating patients with significant respiratory disease and compromised lung function with a reduced dependence on patient inspiratory flow for delivery compared to other inhaler devices [18]. This results in these target patients receiving enhanced delivery and improved disease outcomes [17]. They are also beneficial for patients with tracheostomies, those requiring mechanical ventilation and paediatric patients [19, 20]. However, nebuliser’s are not without their limitations; in addition to their often cumbersome and noisy nature, nebulizer technologies including jet and ultrasonic nebulizers are unsuitable for the aerosolisation of some biomolecules with issues associated with both delivery efficiency and biomolecule stability [21]. Vibrating mesh nebulizers (VMNs) which are the focus of this study are aerosol generators that convert a liquid drug solution or suspension into an aerosol. VMNs however, offer a potential means of nebulizing biomolecules effectively without affecting stability [22]. The selected devices in this study are particularly suitable for delivery of biomolecules such as IFN-γ as less stress is put on the therapeutic cargo than that imposed by a jet or ultrasonic nebulizer. Ultrasonic and jet nebulizers may damage protein and other complex agents through heat or shear stress [23].

Studies to date have focused on the safety and efficacy profile of nebulized IFN-γ but not on the aerosol properties or pharmaceutical characteristics including stability of the IFN-γ post-nebulization. Nebulizers used include the Respirgard II jet nebulizer [12], the PARI BOY N® jet nebulizer [24], and the ultrasonic I-neb Adaptive Aerosol Delivery System [25]. Here, we aerosolized IFN-γ using a commercially available vibrating-mesh technology (Aerogen Solo) and a novel mesh technology (PDAP, also developed by Aerogen). The novel PDAP device was designed to facilitate both low droplet size and faster flow rates, allowing potentially for more precise delivery to specific target areas within the airways. This unique combination of higher output rate and lower droplet size could potentially reduce treatment times whilst enabling deposition of a greater portion of the therapeutic aerosol in the lower airways. Herein we challenge the utility of this novel technology in combination with IFN-γ, a classically difficult-to-deliver biomolecule cargo of clinical interest, and compare its performance to the current mesh technology, embodied in the Aerogen Solo, and already reported as used in the delivery of IFN-γ.

In order to determine the utility of both iterations of vibrating mesh technology for IFN-γ aerosolisation, extensive aerosol performance assessments were completed which included; delivered dose, post-nebulization stability and activity, MMAD, VMD and output rate (ml/min).

## Materials and methods

### Interferon Gamma

Immukin® (IFN-γ), Boehringer Ingelheim Immukin was purchased from Uniphar Group, Dublin, Ireland and used in all experimental work. Human IFN-γ enzyme-linked immunosorbent assay (ELISA) MAX™ Deluxe kit and Human CXCL 10 (IP 10) ELISA MAX™ were obtained from BioLegend, San Diego, CA 92121. All other materials unless specified were purchased from Sigma Aldrich, Ireland.

### Vibrating mesh Nebulisers (VMN)

**Fig. 1:**
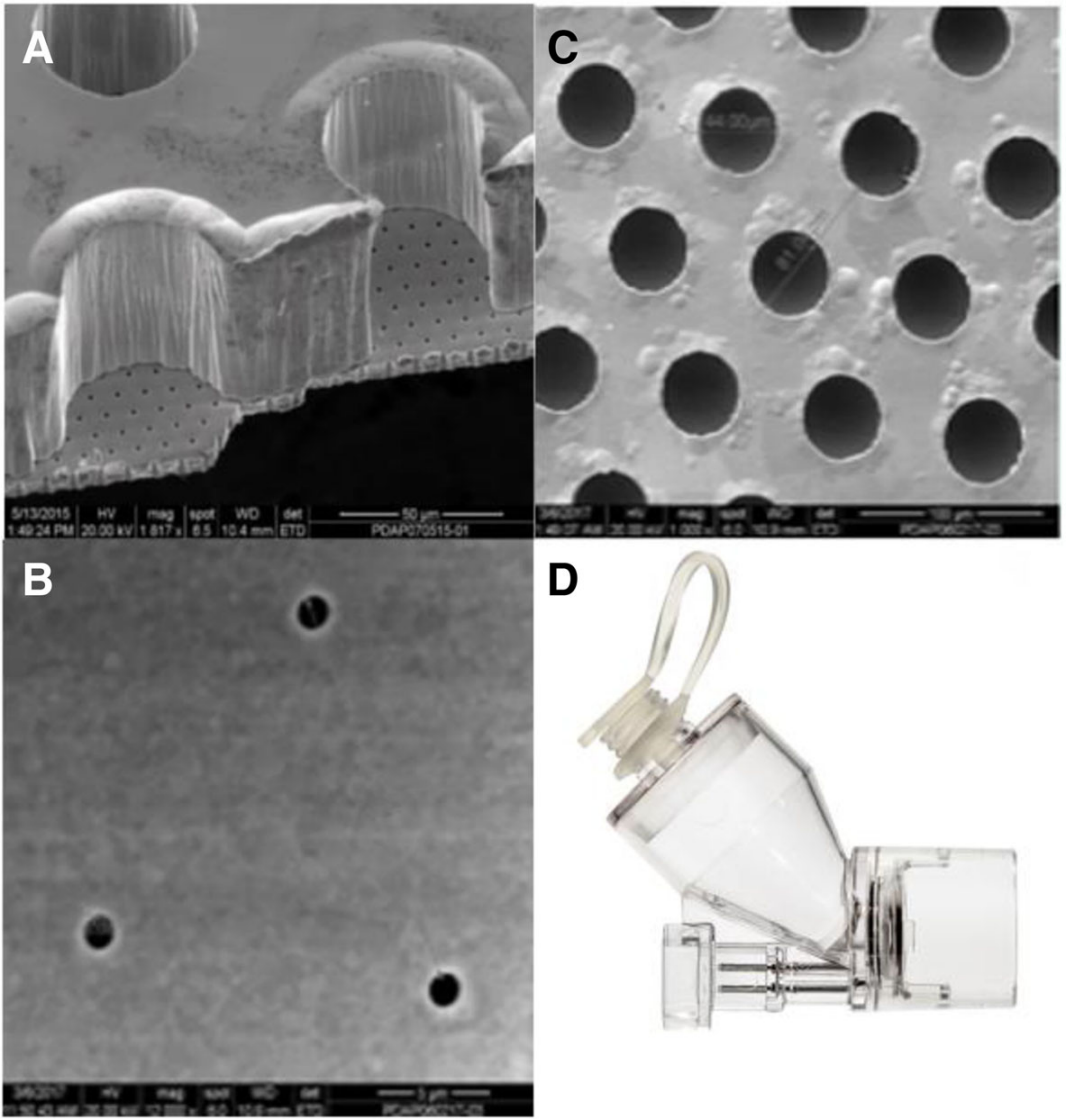
Mesh architecture: **a** General perspective view of reservoir wells on a thick support layer overlaid on a thin outlet layer as captured by scanning electron microscope (SEM) (Magnification 817x). **b** Reservoir image of the PDAP (photo defined aperture plate) device as captured by SEM. **c** Outlet holes of the PDAP device aperture plate as captured by SEM. **d** Solo and PDAP mesh housing

Two vibrating mesh-type nebulizers (Aerogen Solo and Aerogen PDAP, Aerogen, Galway, Ireland) were assessed in this study. The Aerogen Solo is a single layer photo-defined vibrating mesh which has a 5 mm aperture plate diameter, perforated with 1000 precision formed holes, vibrating at 128,000 times per second. This mesh produces droplets 1–5 μm in diameter and is made from Nickel/Palladium mix. The overall thickness is ~ 60 μM. The Aerogen PDAP is a photo defined 2-layer vibrating mesh, also produced using a Nickel/ Palladium mix. It also has a 5 mm aperture plate diameter (defined by the physical properties as demonstrated in Fig. 1 below) with 15,800 holes, also vibrating at 128,000 times per second.

### Emitted dose characterization

A known concentration (500, 250, 125 pg/mL) in 1 ml IFN-γ was nebulized using either the Aerogen Solo or PDAP mesh technology. The respective aerosols produced were captured directly in a sealed 50 mL tube. Following complete nebulization of the dose, the device was powered off, removed and the tube centrifuged (Hettich Rotina 35R Bench centrifuge, 5 mins, 10,000 rpm) in order to pool the sample prior to refrigeration at 4-8 °C. The concentration of delivered dose was determined using a Human IFN-γ ELISA MAX™ Deluxe kit (BioLegend, San Diego, CA 92121) as per kit instructions. The medication cup of both the Solo and PDAP were washed and residual volume calculated.

### Aerosol output rate and droplet size characterization

VMD and aerosol output rate were determined using laser diffraction (Malvern Spraytec, Malvern, UK) as previously described [26]. The MMAD of each aerosol was also determined by cascade impaction at 15  litres per minute (LPM) (Next Generation Impactor (NGI), Copley Scientific, UK) [27]. For each replicate, a fixed volume of 0.5 mL of 6.25 μg/ml IFN-γ was aerosolized. Gravimetric measures were used to determine the distribution of aerosol droplets across the impactor stages. Prior to and post runs, nebulizers and NGI cups were weighed using an electronic analytical balance (OHAUS Pioneer®, Switzerland). The MMAD, and geometric standard deviation (GSD) were calculated using validated software (CITDAS version 3.10 Copley, UK). Triplicate runs were performed for each device.

### Simulated aerosol delivery during a non-invasive patient intervention – spontaneous breathing

**Fig. 2:**
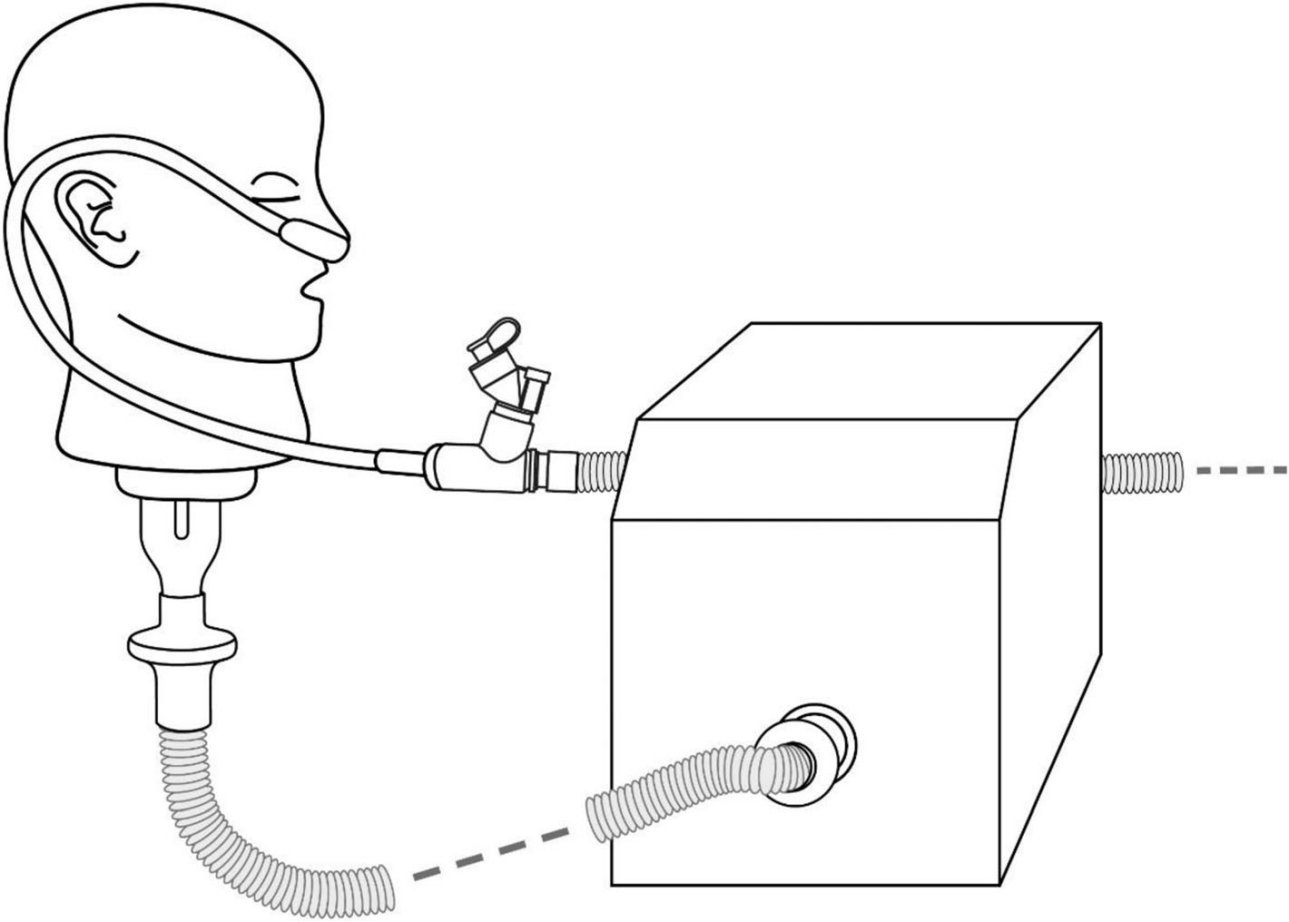
Tracheal Dose test setup with high flow nasal cannula, showing the adult head model attached to the breathing simulator

Non-invasive Nasal High Flow therapy is increasingly used across a variety of patient populations and so it may be beneficial to combine this intervention with inhaled drug therapy including biomolecules [28]. Investigations by Branconnier and colleagues [29] determining non-invasive nasal high flow therapy-nebulizer compatibility employed albuterol sulphate as an exemplar drug, as have studies by Diaz and colleagues [25], and our own colleagues [30]. We chose to use albuterol as a tracer aerosol (see study limitations). Albuterol is a commonly nebulized bronchodilator, regularly reported in the aerosol therapy literature as a means of aerosol performance characterization. Albuterol sulphate is also specified for use as a tracer aerosol in the international standard ISO 27427:2013. The Ingmar ASL 5000 breathing apparatus was used to simulate a healthy adult (15 BPM, Vt 500ML, I/E 1:1) [31]. An adult high flow nasal therapy system (Optiflow™, Fisher and Paykel) was attached to a humidifier (MR850™, Fisher and Paykel) set at 37 °C. The nebulizers were placed at the humidifier, in line with previous findings for maximal aerosol delivery during high flow therapy [30]. 2 ml of 2 mg/ml Albuterol sulphate solution was nebulized by both the Aerogen Solo and PDAP mesh devices. Tracheal Dose [Dose delivered beyond the trachea] at each gas flow rate under test (10, 30, 45 LPM) was recorded on an absolute filter (Respirgard 303, Baxter) distal to the adult airway model (*n* = 3) as shown in Fig. 2. The mass of drug eluted from the filters was determined using UV spectroscopy (276 nm) and interpolation on a standard curve of albuterol sulphate concentrations.

### Simulated aerosol delivery during an invasive patient intervention – mechanical ventilation

In line with the non-invasive delivery modality investigations, albuterol sulphate served as a surrogate drug for IFN-γ during simulated invasive mechanical ventilation via an endotracheal tube. The Maquet Servo-i ventilator was used to generate an adult breathing pattern (Volume Control Ventilation, 15 BPM, 500 ml V_t_, I:E 1:2, PEEP 5cmH20) with a 22 mm endotracheal tube. Albuterol sulphate (2 ml of 2 mg/ml solution) was nebulized by both the Aerogen Solo and PDAP devices. Both the Aerogen Solo and PDAP nebulizers were placed between the wye and endotracheal tube (Flexicare, 038–961-080, ID 8.0 mm, UK). The inhaled dose was recorded on an absolute filter (Respirgard 303, Baxter, Ireland) distal to the ETT (*n* = 3). The mass of drug eluted from the filters was again determined using UV spectroscopy (276 nm) and interpolation on a standard curve of albuterol sulphate concentrations.

### Bioactivity assay

Electrophoresis was carried out using XCell SureLock® NuPAGE Bis-Tris Mini Gels 4–12%. Protein samples (25 μg/ml) were prepared as per manufacturer guidelines.

CXCL 10, also known as IP 10 is a key biomarker in many disease states and plays a significant role following IFN-γ induced release, in many inflammatory conditions including those affecting the pulmonary system. There is a positive feedback loop between IFN-γ release by ThP-1 cells and IP10 release thus interruption of the loop can alter the inflammatory response produced [32]. ThP-1 cells stimulated with IFN-γ release IP10 and as such can be used to determine the bioactivity of nebulized IFN-γ. ThP-1 cells were grown in RPMI 1640 media containing 10% foetal bovine serum supplemented with 100 μg/ml penicillin, 100 μg/ml streptomycin and 2 mmol/l L-glutamine and maintained in a 5% CO_2_ incubator at 37 °C.

For the bioactivity assay of post-nebulized IFN-γ, ThP-1 cells were seeded at a density of 1X10^5^ cells/ml in a 24 well plate and differentiated for three days with phorbol myristate acetate (PMA) prepared as a stock solution (10 mM) in dimethyl sulfoxide and diluted to a final concentration 100 nM in media. Following differentiation media was removed and replaced with media containing a known concentration (20, 10, 5 ng/ml) of non-nebulized (control) and nebulized IFN-γ. After 24 h the supernatants were collected and the amount of released CXCL 10 (IP 10) was measured by ELISA (Human CXCL 10 (IP 10) ELISA MAX™ Deluxe kit) using the ELISA kit protocol.

### Statistical analysis

Data were expressed as mean ± standard error. All statistical analyses were conducted using.

GraphPad Prism 5 software (GraphPad, San Diego, CA, USA). Data were analyzed by a two-way Anova. A *P* value of less than 0.05 was deemed significant.

## Results

### Emitted dose

**Fig. 3:**
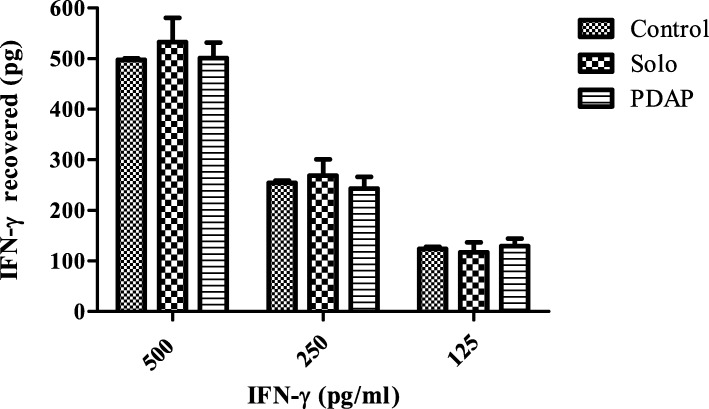
IFN-γ recovery (pg) of 1 ml dose volumes of known doses of IFN-γ (500–125 pg/ml) using both VMNs (vibrating mesh nebulizer’s) - Solo and PDAP devices. Results were compared to non-nebulized controls (*n* = 6)

IFN-γ at three differing concentrations (500, 250, 125 pg/ml) was quantified postnebulization using the Solo and PDAP devices and compared to non-nebulized controls. The amount of IFN-γ emitted by each device was determined by ELISA, as previously described. Non-nebulized controls were assayed for each concentration and as shown in Fig. 3 proved to be 101.80% (509.28 pg/ml ± 30.78), 97.22 (243.04 pg/ml ± 23.30) and 103.47% (129.34 pg/ml ± 15.062) recovered for each of the doses 500, 250, and 125 pg/ml respectively (*n* = 6). There was no significant difference in the amount of IFN-γ retrieved post-nebulization using the either the Solo (99.59%: 497.96 pg/ml ± 2.901, 101.62%: 254.042 pg/ml ± 4.74 and 99.07%: 123.84 pg/ml ± 3.66) or PDAP devices (106.59%: 532.97 pg/ml ± 47.858, 107.60%: 268.99 pg/ml ± 31.584 and 93.81%: 117.26 pg/ml ± 19.37). Thus it can be concluded that nebulization using either device results in at least 93% dose delivery for IFN-γ and in terms of dose delivery the PDAP matches closely that achieved with the commercially available Solo device. With the balance remaining as residual within the medication cup.

### Aerosol output and droplet size characterization

Both mesh iterations were investigated for their ability to aerosolize a 0.5 ml dose of 6.25 μg/ml (chosen originally due to detection limits of ELISA plates) of IFN-γ with droplet size and aerosol output rates. Copley software facilitated calculation of MMAD and GSD of nebulized droplets (results shown in Table 1). The output rate proved similar for the test VMNs (PDAP: 0.44 ml/min in comparison with 0.42 ml/min for Aerogen Solo) however droplet size differed significantly between the devices with VMD and MMAD values for Aerogen Solo almost twice those produced but the PDAP. In both cases however, the nebulizers produced IFN-γ droplets suitable for pulmonary delivery with droplets falling within the optimal MMAD/ respirable range of 0.5–5 μm [16]. The PDAP device however, may be more suitable for drug delivery to the lower airways as both the VMD and MMAD indicate that it produces significantly smaller droplets 2.33 μm, 2.79 μm with GSD 1.47 μm respectively in comparison to 4.39 μm, 4.30 μm and GSD 1.90 μm for Aerogen Solo. In Table 1, *P*-values of < 0.0001 for VMD and 0.0005 for MMAD indicate that a statistical difference for both MMAD and VMD exists between the two devices.

**Table 1:** The volume median diameter (VMD), mass mean aerodynamic diameter (MMAD) and geometric standard deviation (GSD) of IFN-γ aerosol droplets produced by the test devices (PDAP and Aerogen Solo). *P*-values < 0.05 were considered significant

Vibrating mesh type	VMD (μm)	MMAD (μm)	GSD (μm) based on MMAD values
PDAP	2.33 ± 0.02	2.79 ± 0.29	1.47 ± 0.07
Solo	4.39 ± 0.02	4.30 ± 0.25	1.90 ± 0.04
*P*-value	< 0.0001	0.0005	N/A

### Aerosol delivery during simulated non-invasive ventilation

The Ingmar ASL 5000 breathing apparatus was used to simulate a healthy adult. Albuterol sulphate was delivered via an adult nasal high flow interface at varying clinically relevant gas flow rates. Within respiratory medicine there is a growing interest in a potential role for IFN-γ in the treatment of IPF, TB and other inflammatory respiratory conditions. This has led researchers to explore direct, targeted delivery of the biomolecule via inhalation, as an alternative route of administration to injection. This patient acceptable, easy to use and non-invasive delivery system offers targeted delivery for respiratory diseases thus improved clinical response with minimal adverse effects [33]. The results of testing are presented in Table 2. Results are expressed as a percentage of the nominal dose placed in the nebulizer medication cup.

**Table 2:** Mean tracheal dose delivered of albuterol sulphate (2 mg/ml) a surrogate drug for IFN-γ using the LUCY adult airway model and test devices (PDAP and Aerogen Solo)(*n* = 3). *P*-values < 0.05 were considered significant

Gas Flow Rate (LPM)	PDAP	Solo	*P*-value
	(%) Tracheal Dose Mean (± STDEV)		
10	27.32 ± 0.47	22.45 ± 0.19	< 0.0001
30	21.35 ± 0.64	15.67 ± 0.45	0.0002
45	9.54 ± 0.85	5.10 ± 0.07	0.0008

The data presented in Table 2 indicates that across all three gas flow rates investigated, the difference between both test devices is significant, with *P*-values of < 0.0001, 0.0002 and 0.0008 at 10, 30 and 45 LPM respectively. The PDAP device delivered a higher tracheal dose for all gas flow rates used in this study. This suggests that a patient would receive more drug than when using the Solo device [7]. It can also be seen that an increase in gas flow rate leads to a reduction in efficiency. This may be due to particle collisions in the circuit and also within the head model itself. MacLoughlin and colleagues [34] previously reported that higher flow rates are associated with reduced efficiency of drug delivery through an adult NHF system.

### Aerosol delivery during simulated invasive mechanical ventilation

The Maquet Servo-i ventilator was used to generate a healthy adult breathing pattern as described in Table 3. Inhaled dose at the end of an 8.0 mm adult ETT was determined. This test is to determine (as a percentage) inhaled dose delivered to the patient in a clinical or hospital setting. The results of testing are presented in Table 3. Results are expressed as a percentage of the nominal dose placed in the nebulizer medication cup.

**Table 3:** Mean Inhaled dose delivered of albuterol sulphate (2 mg/ml) a surrogate drug for IFN-γ using adult breathing parameters during mechanical ventilation and test devices (PDAP and Aerogen Solo) (*n* = 3). *P*-values < 0.05 were considered significant

PDAP	Solo	*P*-value
Inhaled Dose Mean (%)(± STDEV)		
24.58 ± 0.21	18.31 ± 0.45	< 0.0001

Table 3, shows a statistically significant difference between the percentage inhaled dose mean for the Aerogen Solo and PDAP at 18.31 and 24.58%, respectively. As with non-invasive therapy the PDAP device is more efficient in this study. This means the patient is inhaling a higher dose when compared to the Solo.

### Protein stability post-nebulization

**Fig. 4:**
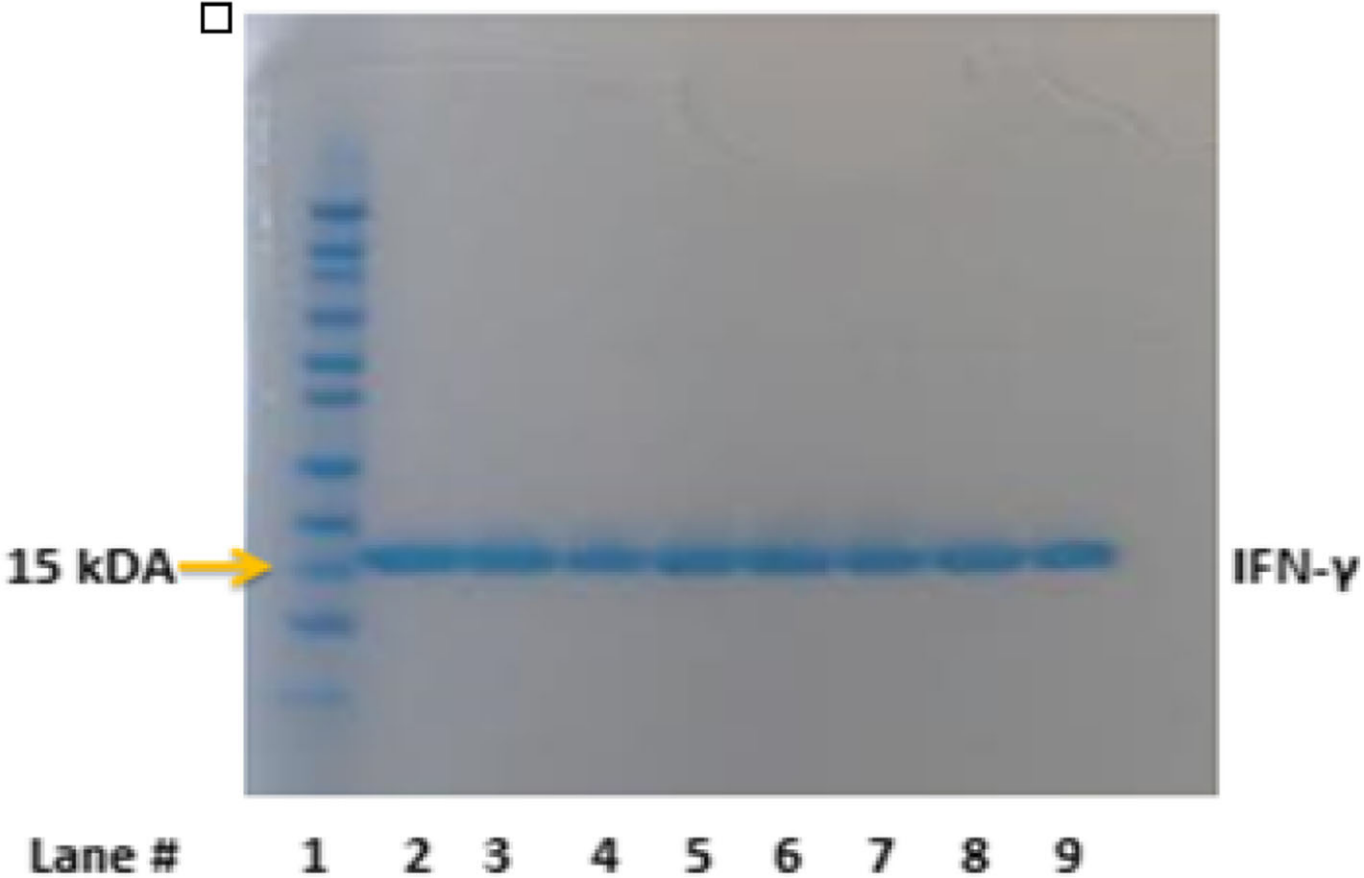
SDS-PAGE analysis indicated that IFN-γ remained intact post-nebulization from both devices Aerogen Solo (lanes 4–6) and PDAP (lanes 7–9) in comparison to non-nebulized controls (lanes 2–3). The molecular weight marker is in lane 1

IFN-γ was assessed for molecular weight and signs of degradation using SDS-PAGE post-nebulization. Three samples nebulized through the Aerogen Solo and three nebulized though the PDAP device were loaded onto a SDS-PAGE gel as shown in Fig. 4 (25 μl of boiled protein 25 μg/ml). Nebulized IFN-γ using Aerogen Solo® (lanes 4, 5 and 6) and PDAP device (lanes 7, 8 and 9) exhibited no changes in molecular weight or detectable degradation compared to non-nebulized controls (lanes 2 and 3) as evidenced by no change in bands on the gel.

### Bioactivity of nebulized IFN-γ

**Fig. 5:**
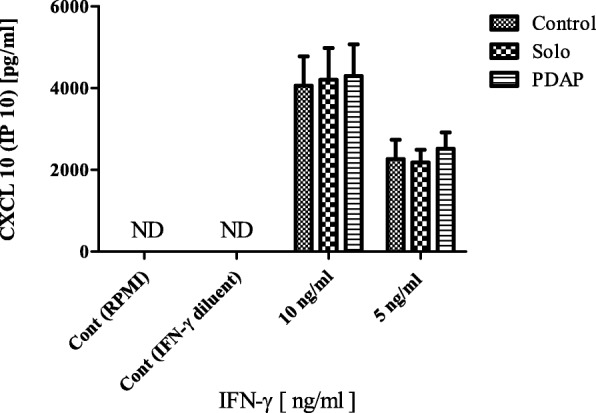
CXCL-10 release from THP-1 cells (*n* = 5) following treatment with nebulized IFN-γ (using Solo and PDAP devices) and non-nebulized (control) IFN-γ at 10 and 5 ng/ml. Two additional controls were employed RPMI and IFN-γ diluent and were non-detectable (ND)

The bioactivity of nebulized IFN-γ was determined using THP-1 cells. Cells were activated using PMA (100 nM) and treated with bioactive IFN-γ. The chemokine CXCL10 subsequently released into cell culture supernatant, the quantity of which was determined by ELISA and validated using non-nebulized IFN-γ. IFN-γ at a range of concentrations, 10 and 5 ng/ml, was nebulized using both devices (Aerogen Solo® and PDAP devices) and exposed to activated THP-1 cells in culture for 24 h. As seen in Fig. 5 (*n* = 5) there was no statistical difference in the amount of CXCL 10 (IP 10) released by THP-1 cells when stimulated with doses of nebulized IFN-γ (10 and 5 ng/ml) from the Aerogen Solo at (4205.75 pg/ml ± 776.859 and 2180.049 pg/ml ± 313.049 respectively) or PDAP device (4295.399 pg/ml ± 779.966 and 2519.717 pg/ml ± 396.657 respectively) in comparison to non-nebulized controls at 5 ng/ml4063.351 pg/ml ± 715.944 and 10 ng/ml 2269.230 pg/ml ± 313.049. RPMI media alone and IFN-γ drug diluent served as controls and there was no detectable CXCL 10 (IP 10) in IFN-γ free supernatants. Bioactivity findings suggest that there is no detrimental effect on the IFN-γ structure and function during nebulization using both vibrating mesh technologies tested and that nebulized IFN-γ effectively stimulated IP 10 release from activated THP-1 cells.

### Study limitations

Although the findings are promising we recognize that there are limitations to our study. Namely, albuterol sulphate was used as a surrogate drug for IFN-γ for both simulated non-invasive and invasive aerosol therapy. In our hands, it was not possible to produce an accurate reliable quantification method for IFN-γ, despite using commercially available kits and following the published literature. It is possible that the IFN-γ was binding to the filter plastics. We also recognize that this is a pre-clinical study and despite our findings indicating effective nebulization of IFN-γ using VMNs it remains to be seen whether these effects are transferable to a clinical environment.

## Discussion

New inhalation devices are enabling the delivery of large biomolecules directly to the lungs. This provides opportunities for the translation of advanced therapies for respiratory disease via the inhaled route. Typically, biologics in the clinic are parenterally administered. Extensive clinical work has been conducted by others regarding the safety and therapeutic efficacy of inhaled IFN-γ [35], however, very limited data is available to-date on the aerosol and pharmaceutical characteristics of nebulized IFN-γ. Such information could inform appropriate or improved device selection and consequently may have a significant effect on clinical outcomes. In this work, we have investigated the performance of two VMNs; a marketed model Aerogen Solo, and a novel Aerogen PDAP device for delivery of IFN-γ.

Aerosolized droplets intended for inhalation must be appropriately sized (1–5 μm) to achieve optimal deposition in the lungs. Smaller aerosols (closer to 1 μm in size) are capable of much deeper delivery, whilst those greater than 5 μm are deposited in the pharynx, and those less than 0.5 μm are likely exhaled prior to deposition [16]. In addition, the recovered dose (100% dose - Residual volume) is an important factor from a therapeutic perspective with a drugs’ efficacy being linked to the properties of both the nebulizer and amount of available aerosolized drug. Our findings indicate that both nebulizers are efficient at delivering at least 93% of a known dose of IFN-γ (500, 250, 125 pg/mL), which is significantly higher than that reported for other nebulizer technologies [36] .

We conducted aerosol output and droplet size analysis to verify that VMNs are capable of delivering IFN-γ at an acceptable rate, and that the aerosols produced are appropriately sized. This builds on work of others relating to aerosolisation of IFN-γ and further supports its potential use as inhaled therapy for inflammatory respiratory conditions such as TB and IPF. Aerosol studies indicated that there was no significant difference between the devices with respect to output rate (PDAP: 0.44 ml/min V Aerogen Solo: 0.42 ml/min). There were however statistically significant differences in aerodynamic particle size distributions for aerosols produced from the two VMNs with the PDAP producing much smaller VMD, MMAD and GSD values (2.33, 2.79 and 1.47 μm respectively). Aerosol droplets of IFN-γ produced by the PDAP had a smaller MMAD than seen in a previous study that nebulized IFN-γ (Immukin) using a PARI jet nebulizer (MMAD 3.7 μm) [24]. Diaz and colleagues [25] have shown that radio-labelling of IFN-γ permits imaging of lung deposition in patients. Radio-labelled IFN-γ nebulized using a VMN (I-neb Adaptive Aerosol Delivery System) produced droplets of similar size to those produced by the PDAP device investigated in our study (2.8 μm). Droplets of this size were suitably sized to reach the middle lobe area in the lungs. The ability of PDAP to generate droplets of a smaller aerodynamic size could offer advantages over currently marketed devices in terms of targeting IFN-γ to the distal airways for treatment of TB and/or IPF.

The surrogate drug albuterol (specified for use as a tracer aerosol in the international standard ISO 27427:2013) was delivered with the Aerogen PDAP and compared to the commercially available Aerogen Solo. For both simulated non-invasive and invasive patient interventions that facilitate concurrent aerosol therapy, the PDAP demonstrates improvements in efficiency for both tracheal and inhaled dose when compared to the Aerogen Solo.

SDS-PAGE indicates that IFN-γ remains structurally intact following nebulization using both vibrating mesh technologies. This also confirms that the droplets produced and measured in NGI studies are aerosolized IFN-γ and not breakdown products. VMNs such as those tested in this study therefore appear to be suitable delivery methods for IFN-γ. Uniquely, the PDAP facilitied a fast output rate (0.44 ml/min) in conjunction with a small droplet size (2.79 μm MMAD), optimal for lung targeting. Further confirmation of drug stability post-nebulization was determined through bioactivity assays. THP-1 cells differentiated into macrophages were used to conduct IFN-γ bioactivity, studies. THP-1 cells are considered as suitable models for determining alveolar macrophage response (including cytokine CXCL10 release) to IFN-γ. Increased CXCL10 levels are associated with many inflammatory pulmonary conditions including asthma thus CXCL10 expression, and receptors, are potential targets for novel therapies including inhaled IFN-γ [37]. Raju and colleagues [38] demonstrated that IFN-γ treated THP-1 cells displayed a similar increase in CXCL10 expression to those exposed to TB infections, and the combination of TB infection and IFN-γ further increased IP10 release. We observed CXCL10 release following exposure of THP-1 cells to control (non-nebulized) IFN-γ. Nebulization using VMNs did not affect the bioactivity of IFN-γ, as there was no statistical difference between CXCL10 release elicited by nebulized IFN-γ and non-nebulized IFN-γ controls. This confirms the potential of both VMN devices (Solo and the PDAP) to deliver therapeutically active IFN-γ via nebulization in an in vitro setting. Efficient delivery of IFN-γ locally to TB and/or IPF patients via nebulization could support a patient-friendly, targeted approach to treatment and potentially improve therapeutic outcomes as shown in clinical studies [24].

## Conclusions

Whilst further clinical evaluation is required, using international standard methodologies for the assessment of nebulizer performance in patients, we have demonstrated the feasibility of an alternative nebulizer type to the commonly reported jet nebulizer type for the aerosol-mediated delivery of IFN-γ pre-clinically. The new iteration of mesh technology, the Aerogen PDAP, serves to improve delivery efficiency of IFN-γ delivery over the current Solo device. Our findings show that the integrity and bioactivity of IFN-γ are maintained post-nebulization using the new VMN. This device therefore offers a rational means of facilitating the clinical translation of inhaled IFN-γ for application in diseases such as IPF and TB.

## Abbreviations

(ELF: epithelial lining fluid
ASL: active servo lung
CGD: chronic granulomatous disease
CXCL 10: C-X-C motif chemokine 10
ELISA: enzyme-linked immunosorbent assay
GSD: gravimetric standard deviation
IFNs: interferons
IFN-γ: Interferon gamma
IL: interleukin
IP 10: IFN-γ-induced protein 10KDa
IPF: idiopathic pulmonary fibrosis
LPM: liters per minute
MMAD: mass median aerodynamic diameter
NGI: next generation impactor
PDAP: photo-defined aperture plate
PMA: phorbol myristate acetate
SEM: scanning electron microscopy
SLE: systemic lupus erythematous
TB: tuberculosis
THP-1 cells: THP-1 derived macrophages
VMD: volume median diameter
VMNs: vibrating mesh nebulizers

## References

[1] Lipiäinen T, Peltoniemi M, Sarkhel S, Yrjönen T, Vuorela H, Urtti A, et al. Formulation and Stability of Cytokine Therapeutics. J Pharm Sci [Internet]. Elsevier; 2015 [cited 2019 Mar 5];104:307–26. Available from: https://www.sciencedirect.com/science/article/pii/S0022354915302161 10.1002/jps.24243 25492409

[2] Schroder K, Hertzog PJ, Ravasi T, Hume DA (2003). Interferon-γ: an overview of signals, mechanisms and functions. J Leukoc Biol.

[3] Miller CHT, Maher SG, Young HA. Clinical use of interferon-γ. Ann N Y Acad Sci Wiley/Blackwell (10.1111); 2009;1182:69–79. 10.1111/j.1749-6632.2009.05069.x PMC6574079 20074276

[4] Lyseng-Williamson KA (2015). Interferon γ-1b in chronic granulomatous disease and severe malignant osteopetrosis: a guide to its use in the USA. Drugs Ther Perspect.

[5] Zaidi MR, Merlino G. The two faces of interferon-γ in cancer. Clin Cancer Res. 2011:6118–24. 10.1158/1078-0432.CCR-11-0482 PMC3186825 21705455

[6] Greenlee-Wacker MC, Nauseef WM. IFN-γ targets macrophage-mediated immune responses toward *Staphylococcus aureus*. J Leukoc Biol Wiley-Blackwell; 2017;101:751–758. 10.1189/jlb.4A1215-565RR PMC5295848 27707882

[7] Smaldone GC (2018). Repurposing of gamma interferon via inhalation delivery. Adv Drug Deliv Rev.

[8] Raghu G, Brown KK, Williamson Z, Bradford WZ, Starko K, Noble PW, Schwartz DA, King TE (2004). A placebo-controlled trial of interferon gamma-1b in patients with idiopathic pulmonary fibrosis. N Engl J Med.

[9] Patton JS. Mechanisms of macromolecule absorption by the lungs. Adv Drug Deliv Rev. 1996:3–36.

[10] Jaffe HA, Buhl R, Mastrangeli A, Holroyd KJ, Saltini C, Czerski D (1991). Organ specific cytokine therapy. Local activation of mononuclear phagocytes by delivery of an aerosol of recombinant interferon-gamma to the human lung. J Clin Invest American Society for Clinical Investigation.

[11] Fusiak T, Smaldone GC, Condos R (2015). Pulmonary fibrosis treated with inhaled interferon-gamma (IFN-γ). J Aerosol Med Pulm Drug Deliv..

[12] Moss RB, Mayer-Hamblett N, Wagener J, Daines C, Hale K, Ahrens R (2005). Randomized, double-blind, placebo-controlled, dose-escalating study of aerosolized interferon gamma-1b in patients with mild to moderate cystic fibrosis lung disease. Pediatr Pulmonol.

[13] Condos R, Hull FP, Schluger NW, Rom WN, Smaldone GC. Regional deposition of aerosolized interferon-gamma in pulmonary tuberculosis. Chest. Elsevier; 2004;125:2146–2155. 10.1378/chest.125.6.2146 15189935

[14] Millard J, Ugarte-, Gil C, Moore. Multidrug resistant tuberculosis BMJ 2015;350–882. 10.1136/bmj.h882 25721508

[15] Condos R, Rom WN, Schluger NW (1997). Treatment of multidrug-resistant pulmonary tuberculosis with interferon-γ via aerosol. Lancet..

[16] Mortensen NP, Durham P, Hickey AJ. The role of particle physico-chemical properties in pulmonary drug delivery for tuberculosis therapy. J Microencapsul. 2014:785–95. 10.3109/02652048.2014.932029 25090595

[17] Ari A, Arı A (2014). Jet, ultrasonic, and mesh nebulizers: an evaluation of nebulizers for better clinical outcomes. Eurasian J Pulmonol Eurasian J Pulmonol.

[18] Ibrahim M, Verma R, Garcia-Contreras L. Inhalation drug delivery devices: technology update. Med Devices Evid Res. 2015:131–9. 10.2147/MDER.S48888 PMC4334339 25709510

[19] Heyder J (2004). Deposition of inhaled particles in the human respiratory tract and consequences for regional targeting in respiratory drug delivery. Proc Am Thorac Soc.

[20] MacLoughlin R, Telfer C, Clark A, Fink J (2017). Aerosol: a novel vehicle for pharmacotherapy in neonates. Curr Pharm Des.

[21] Daniel RM, Cowan DA. Biomolecular stability and life at high temperatures. Cell Mol Life Sci. 2000:250–64. 10.1007/PL00000688 PMC11146935 10766021

[22] de Kruijf W, Ehrhardt C. Inhalation delivery of complex drugs — the next steps. Curr Opin Pharmacol. 2017:52–7. 10.1016/j.coph.2017.07.015 28846876

[23] O’Riordan TG (2002). Formulations and nebulizer performance. Respir Care.

[24] Grahmann PR, Braun RK (2008). A new protocol for multiple inhalation of IFN-γ successfully treats MDR-TB: a case study. Int J Tuberc Lung Dis.

[25] Diaz KT, Skaria S, Harris K, Solomita M, Lau S, Bauer K, et al. Delivery and safety of inhaled interferon-γ in idiopathic pulmonary fibrosis. J aerosol med Pulm drug Deliv. Mary Ann Liebert, Inc. 140 Huguenot street, 3rd floor New Rochelle, NY 10801 USA ; 2012;25:79–87. 10.1089/jamp.2011.0919 22360317

[26] O’Mahony AM, Nolan L, O’Driscoll CM, Hibbitts A, Desgranges S, Cryan SA (2014). Early-stage development of novel Cyclodextrin-siRNA Nanocomplexes allows for successful Postnebulization transfection of bronchial epithelial cells. J Aerosol Med Pulm Drug Deliv.

[27] United States pharmacopeia and National Formulary (USP 29-NF 24). 601 aerosols, nasal sprays, metered-dose inhalers, and dry powder inhalers.

[28] Dugernier J, Hesse M, Vanbever R, Depoortere V, Roeseler J, Michotte JB (2017). SPECT-CT comparison of lung deposition using a system combining a vibrating-mesh nebulizer with a Valved holding chamber and a conventional jet nebulizer: a randomized cross-over study. Pharm Res.

[29] Branconnier MP, Hess DR (2005). Albuterol delivery during noninvasive ventilation. Respir Care.

[30] Bennett G, Joyce M, Sweeney L, MacLoughlin R (2018). In vitro determination of the Main effects in the Design of High-Flow Nasal Therapy Systems with respect to aerosol performance. Pulm Ther.

[31] Hart N, Polkey MI, Clément A, Boulé M, Moxham J, Lofaso F (2002). Changes in pulmonary mechanics with increasing disease severity in children and Young adults with cystic fibrosis. Am J Respir Crit care med. American thoracic. Society.

[32] Liu M, Guo S, Hibbert JM, Jain V, Singh N, Wilson NO (2011). CXCL10/IP-10 in infectious diseases pathogenesis and potential therapeutic implications. Cytokine Growth Factor Rev Pergamon.

[33] Hickey AJ, Durham PG, Dharmadhikari A, Nardell EA (2016). Inhaled drug treatment for tuberculosis: past progress and future prospects. J Control Release.

[34] MacLoughlin R, Power P, Wolny M, Duffy C (2013). Evaluation of vibrating mesh nebulizer performance during nasal high flow therapy. J Aerosol Med Pulm Drug Deliv.

[35] Martin RJ, Boguniewicz M, Henson JE, Celniker AC, Williams M, Giorno RC (1993). The effects of inhaled interferon gamma in Normal human airways. Am Lung Assoc.

[36] Wan G-H, Fink JB, Lin H-L, Chen Y-H, Liu W-Q, Liu K-Y (2012). Influence of nebulizer type with different pediatric aerosol masks on drug deposition in a model of a spontaneously breathing small child. Respir Care.

[37] Brightling CE, Ammit AJ, Kaur D, Black JL, Wardlaw AJ, Hughes JM (2005). The CXCL10/CXCR3 Axis mediates human lung mast cell migration to asthmatic airway smooth muscle. Am J Respir Crit care med. American thoracic. Society.

[38] Raju B, Hoshino Y, Kuwabara K, Belitskaya I, Prabhakar S, Canova A (2004). Aerosolized gamma interferon (IFN-γ) induces expression of the genes encoding the IFN-γ-inducible 10-Kilodalton protein but not inducible nitric oxide synthase in the lung during tuberculosis. Infect Immun.

